# Changes in the Chemical Barrier Composition of Tears in Alzheimer’s Disease Reveal Potential Tear Diagnostic Biomarkers

**DOI:** 10.1371/journal.pone.0158000

**Published:** 2016-06-21

**Authors:** Gergő Kalló, Miklós Emri, Zsófia Varga, Bernadett Ujhelyi, József Tőzsér, Adrienne Csutak, Éva Csősz

**Affiliations:** 1 Department of Biochemistry and Molecular Biology, Faculty of Medicine, University of Debrecen, Egyetem ter 1., 4032 Debrecen, Hungary; 2 Department of Nuclear Medicine, Faculty of Medicine, University of Debrecen, Egyetem ter 1., 4032 Debrecen, Hungary; 3 Department of Psychiatry, Faculty of Medicine, University of Debrecen, Egyetem ter 1., 4032 Debrecen, Hungary; 4 Department of Ophthalmology, Faculty of Medicine, University of Debrecen, Egyetem ter 1., 4032 Debrecen, Hungary; Pacific Northwest National Laboratory, UNITED STATES

## Abstract

Alzheimer’s disease (AD) is one of the most common neurodegenerative diseases, with increasing prevalence affecting millions of people worldwide. Currently, only autopsy is able to confirm the diagnosis with a 100% certainty, therefore, biomarkers from body fluids obtained by non-invasive means provide an attractive alternative for the diagnosis of Alzheimer`s disease. Global changes of the protein profile were examined by quantitative proteomics; firstly, electrophoresis and LC-MS/MS were used, thereafter, SRM-based targeted proteomics method was developed and applied to examine quantitative changes of tear proteins. Alterations in the tear flow rate, total tear protein concentration and composition of the chemical barrier specific to AD were demonstrated, and the combination of lipocalin-1, dermcidin, lysozyme-C and lacritin was shown to be a potential biomarker, with an 81% sensitivity and 77% specificity.

## Introduction

Alzheimer’s disease (AD) is one of the most common forms of age-related dementia, affecting more than 25 million people worldwide, with the number of new cases being continuously on the rise, particularly in the developed and developing countries [1]. The etiology of AD is still unknown. The disease can be characterized by massive cognitive decline, occurring in either sporadic or familial forms. Evidence suggests that abnormal production and accumulation of misfolded, toxic proteins like β-amyloid (Aβ) peptides, the microtubule-associated protein tau [2], and the presynaptic protein α-synuclein are involved in the pathogenesis of AD [3]. The pathological hallmarks of AD are the appearance of senile plaques and neurofibrillary tangles in the brain tissue, in addition to the development of cerebral amyloid angiopathy, with depositions of Aβ peptides in the vessel walls [4].

Based on reported data, AD affects the entire visual system; AD-related changes have been observed in the eye, the visual pathway, as well as the visual cortex. Aβ depositions in the lens and retina were also detected, along with changes such as the reduction of the numbers of retinal ganglion cells, thinning of the nerve cell layer, optic atrophy and decline in the numbers of axons affecting mainly the large diameter axons in the optic nerve [5,6]. These changes result in the impairment of visual acuity and color vision, defects in fixation, delay in saccadic eye movement and changes in contrast sensitivity [7].

Multiple studies highlighted the role of Aβ and hyperphosphorylated tau [8] in the pathophysiology of the disease, however, new evidences suggest the involvement of inflammatory processes [9] and the increased oxidation of brain proteins [10], besides the amyloid depositions and neurofibrillary tangle formation. The oxidation of brain proteins is probably the consequence of higher reactive oxygen species (ROS) production that can be caused by inflammatory processes, or by mitochondrial dysfunction, which is often associated with the appearance of AD [11].

Several biomarkers for AD were identified, yet an ideal biomarker that is able to improve differential diagnosis, track disease progression and measure treatment efficacy has not been discovered. Despite advances in neuroimaging techniques [12,13], and the opportunities provided by the “omics” and systems biology studies [14], autopsy remains the only mean to provide diagnosis with a 100% certainty. Genetic markers; such as mutations in the gene of amyloid precursor protein (APP), presenilin 1 and 2 (PS1 and PS2) and tau, were identified for early onset AD, while apolipoprotein E (APOE) and clusterin gene (CLU) mutations were identified for the late onset form [15,16]. Disease-specific mutations indicating the alteration of fatty acid and amino acid metabolism, innate immune response, apoptosis, cell communication, and cell adhesion pathways in the brain of AD patients were also highlighted [17]. For biomarker analyses, brain tissue, cerebrospinal fluid (CSF), blood plasma and serum were used, and several candidate protein biomarkers were identified using advanced proteomics techniques, such as two dimensional gel electrophoresis, oxyblotting, mass spectrometry, and multiplex assays [14,18].

Selected reaction monitoring (SRM)-based targeted proteomics analysis is a versatile tool for biomarker studies. SRM is a special scan mode of the triple quadrupole-containing mass spectrometers, which allows for highly specific identification; and with the help of stable isotope-labeled (SIL) synthetic peptides, it enables the relative or absolute quantification of the analytes of interest [19,20]. Another advantage of SRM is the multiplex feature, multiple molecules can be analyzed simultaneously from the same sample, which allows for a more cost-effective analysis compared to the classical antibody-based quantification techniques [21].

With increasing AD prevalence, there is a high demand for novel diagnostic methods utilizing body fluids collected by non-invasive means, such biomarkers are of valuable importance. Tear fluid; therefore, provides a viable source, and with its relatively high protein content [22], it is a widely used candidate for biomarker studies [23–25]. Tear samples are easy to collect, thus, they provide excellent sample source for developing bedside diagnostic tests for pathological conditions affecting the ocular system, or the body as a whole [26,27]. The tear fluid creates a chemical barrier at the surface of the eye as part of the innate immune system, which provides protection against pathogens by secreting antibacterial and immunomodulatory proteins (AMPs) that inhibit bacterial growth [28]. The major tear proteins; such as lipocalin-1, lactotransferrin and lysozyme-C are involved in the immune and inflammatory processes and defense against pathogens [22]. The relatively high abundance of these proteins makes them the major AMPs of tears. Besides the major AMPs, tear fluid contains several prototypic AMPs, such as defensins, LL-37 cathelicidin in addition to dermcidin [22].

Tear fluid was used as a potential biomarker source in a study of neuroinflammation in Parkinson’s disease; a neurodegenerative condition associated with inflammation, and increased TNF-α levels were found in tears of PD patients [29].

Considering the extensive influence of AD on the visual system, in this pilot study, we wanted to examine the changes of tear production, and tear protein composition in patients with AD and in their age-matched controls. A two-step procedure was applied; firsty, gel-based LC-MS/MS was used for protein identification on a small sample size, thereafter, an SRM-based targeted proteomics method was developed for the identified proteins, in order to examine the AD-dependent alterations on a larger sample size, in order to identify possible tear biomarkers for AD.

## Methods

### Sample collection

In total, 23 donors were recruited in this study; 14 patients with AD and 9 age-matched controls. Sample collection complied with the guidelines of the Helsinki Declaration and ethical approval was obtained from the University of Debrecen Ethics Committee (2980–2009), while the subjects gave informed written consent. In the case of AD patients, the consent was acquired in the presence of a caregiver, although none of them were placed under guardianship. All of the donors were patients of the University of Debrecen, Faculty of Medicine, and the assessment of AD was done by a psychiatrist. Diagnosis of Alzheimer’s disease was based on the NINCDS-ADRDA [30] and the DSM-IV-TR [31] criteria. Besides psychiatric and neurological assessment, patients underwent general medical examination, basic laboratory testing (blood chemistry analysis, complete blood cell count, hepatic and renal function test, vitamin B_12_ and folate level determination, thyroid function test) and CT or MR imaging of the brain to rule out other causes of dementia. Patients with a history of sudden onset, early extrapyramidal signs, early behavioral changes or focal neurological features were excluded. The clinical evaluation of the age-matched control subjects consisted of a structured interview, demographic information, medical history, current medication, history of alcohol consumption and a subjective assessment of memory problems using the Mini-Mental State Examination [32]. Only controls without any signs of cognitive impairment were included in the study. Patients with autoimmune disorders, systemic inflammation or ophthalmological disorders were excluded. In case of AD group, the women to men ratio was approximately 1:2 and the mean age was 77 years, whereas in the control group, the women to men ratio was approximately 1:1 and the mean age was 72 years. After assessment of the anterior ocular status of each patient, tear was collected from the inferior meniscus of one or both eyes. The non-traumatic tear collection was carried out without topical anesthesia using standard capillary collection technique [33], thereafter, collected samples were frozen and stored at -70°C until analysis.

### SDS-PAGE

The protein concentration of each tear sample was measured using the Bradford method [34]. 20μg tear protein from three randomly selected AD patients and two controls were subjected to SDS-PAGE analysis on a 10% SDS polyacrylamide gel. Electrophoresis was carried out in a Bio-Rad mini tetra cell (Bio-Rad) on 100 A constant current for one hour. The protein bands were stained using Coomassie PageBlue (Fermentas) solution and scanned with a Pharos FX Plus laser scanner (Bio-Rad). Image analysis was done using the QuantityOne software (Bio-Rad). Band intensities in each case were determined and statistically analyzed by Mann–Whitney U test using SigmaPlot 12.0 software (Systat Software Inc.).

### LC-MS/MS analysis

Bands with significantly different intensities between AD and control samples were excised, followed by in-gel digestion with trypsin. Initially, reduction was performed using 20mM dithiothreitol for one hour at 56°C, followed by alkylation with 55mM iodoacetamide for 45 minutes. Overnight trypsin digestion was carried out using stabilized MS grade TPCK-treated bovine trypsin (ABSciex) at 37°C, thereafter; the digested peptides were extracted and lyophilized. The peptides were re-dissolved in 10μl 1% formic acid before LC-MS/MS analysis.

Prior to mass spectrometric analysis, peptides were separated using a 90 minute water/acetonitrile gradient, with an increase in acetonitrile concentration from 0 to 100% in a 60 minute time interval, using an EasynLCII (Bruker) nano HPLC. The peptide mixture was desalted on a Zorbax 300SB-C18 in-line trap column (5 x 0.3 mm, 5 μm pore size, Agilent), followed by separation on a Zorbax 300SB-C18 analytical column (150 mm x 75 μm 3.5 μm pore size, Agilent). Solvent A was 0.1% formic acid in LC water, solvent B was LC acetonitrile containing 0.1% formic acid, while the flow rate was 300nl/min.

Positive mode LC-MS/MS scans were performed on a 4000 QTRAP (ABSciex) mass spectrometer using a NanoSpray II MicroIon source, controlled by the Analyst 1.4.2 software (ABSciex). The spray voltage was 2800V, the ion source gas was 50 psi, the curtain gas was 20 psi and the source temperature was 70°C. Information Dependent Acquisition method was utilized; after the first mass scan (mass range 400–1700 amu), an enhanced resolution experiment was carried out to establish the charge state of the precursor ions. The MS/MS spectra of the two most intensive ions were recorded (mass range 100–1900 amu) in Enhanced Product Ion mode at a scan rate of 4000 amu/s, and rolling collision energy was applied with the maximum of 90eV.

The acquired LC-MS/MS data were used for protein identification with the help of ProteinPilot 4.0 (ABSciex) search engine searching the SwissProt database (release: 2015.07, 548872 sequence entries), and using the biological modification table included in the ProteinPilot 4.0 software. A minimum of two peptide sequences with ≥95% confidence were used for protein identification.

### Development of SRM-based targeted proteomics experiment

The amino acid sequences of lipocalin-1, lactotransferrin, extracellular glycoprotein lacritin, lysozyme-C, lipophilin A, Ig λ-chain C region, prolactin inducible protein, Zn-α2 glycoprotein, galectin 3 binding protein and dermcidin were retrieved from the UniProt database (P31025, P02788, Q9GZZ8, P61626, P60201, P0CG04, P12273, P25311, Q08380, P81605, respectively), *in silico* trypsin digestions were then carried out. In order to determine the unique tryptic sequences, BLASTp analyses were performed (http://blast.ncbi.nlm.nih.gov), searching the NCBI non-redundant protein sequence database for unique, protein-specific sequences. The protein specific sequences were subjected to SRM transition design using the Skyline software (www.brendanx-uw1.gs.washington.edu). Stable isotope-labeled (SIL) synthetic crude peptides were obtained from JPT Peptide Technologies GmbH, Germany, and the SRM spectra of all singly charged “y” ions were registered in case of all peptides. The best two transitions for each peptide were selected for further analyses.

### SRM-based targeted mass spectrometry analysis

37 tear samples originating from 14 AD patients and 9 age-matched controls were denatured using 6 M urea for 30 mins at room temperature, followed by a digestion procedure similar to that used for LC-MS/MS sample preparation described above. In the denatured, reduced, and alkylated samples, the urea concentration was diluted with 25mM ammonium bicarbonate to 1M immediately before trypsin digestion. Samples were desalted with C18 PierceTips (Thermo Scientific), lyophilized and re-dissolved in 1% formic acid.

SRM-based analyses of digested tear samples were carried out in triplicates on the 4000 QTRAP mass spectrometer using the same voltage, gas, and temperature, as described above in section LC-MS/MS analysis. The declustering potential (DP) and collision energy (CE) values were optimized, and the cycle time was set to 2.5 sec. Chromatographic separation was done on the EasynLC II, using a 30 min acetonitrile/water gradient with an increase in acetonitrile concentration from 0 to 100% during a 15 minute time interval.

Sample blocking was carried out before analyzes; a randomly selected AD sample was paired with a randomly selected control sample and analyzed one after the other using the same conditions. SIL peptides were added to the samples immediately before the analyses. Data were evaluated using the Skyline software, spectra were examined manually and the AUC values were calculated by the software. The SRM data were uploaded to the Panorama website: (https://panoramaweb.org/labkey/project/__r1225/begin.view?) and are publicly available.

The primary AUC data were transformed to MSstats R-package format [35] by an in-house developed software. After the normalization based on the SIL standard peptides, and log2 transformation of data, group differences were examined by a mixed-effect variance analysis [36]. Groups were modeled as fixed effect, while the subject level variances were modeled as random effects. After analysis, the raw p-values were adjusted by the Benjamini and Hochberg type false discovery rate method for multiple testing purposes [37]. Besides the adjusted p-values, the log2 fold change, the standard error and the t-values were also examined. Receiver-operating characteristic (ROC) analyses [38] were carried out by the pROC software [39] and Confidence Interval (CI) values were calculated.

## Results

### Tear protein profile changes in AD

**Fig 1:**
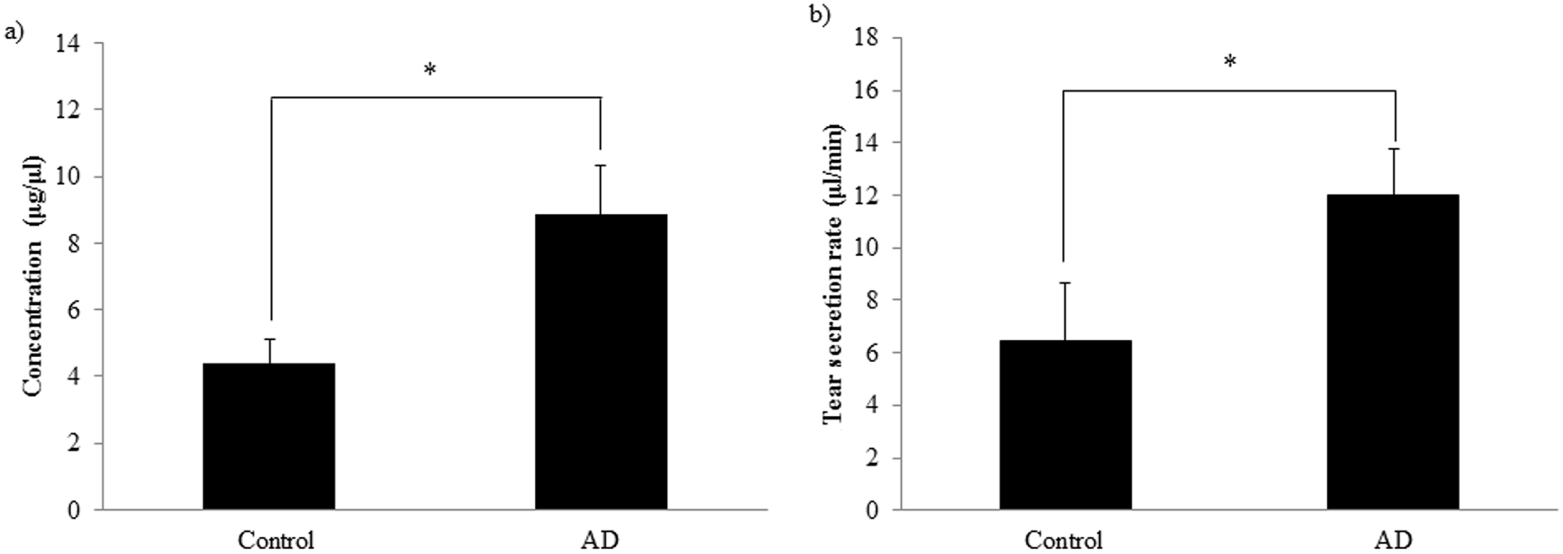
Protein concentration and flow rate of collected tear. The bars represent mean values with the standard error of mean of a) total protein concentration measured by Bradford method b) tear secretion rate. * indicates p <0.05.

The amount of proteins in biological fluids; especially in tear, can provide information of diagnostic relevance. The typical protein concentration in tear fluid is 5–7 μg/μl [40], and the tear production rate is approximately 2 μl/min. Monitoring the tear production rate and tear protein concentration during sample collection (Table 1), significant differences were observed in case of AD patients compared to controls. The flow rate of 6 ± 2 μl/min observed in controls was significantly increased in AD patients (12±2 μl/min), along with a significant increase in tear protein concentration from 4.4 ± 1.4 μg/μl in controls to 8.8 ± 2.9 μg/μl in AD patients (Fig 1).

**Table 1:** Patient data and tear collection parameters.

Patient Nr.	Age (years)	Gender	Tear flow rate (μl/min)	Tear protein concentration (μg/μl)
AD 1	67	M	OD: 10.0	OD:7.1
AD 2	77	M	OD: 12.4 OS: 16.7	OD:3.6 OS:8.3
AD 3	80	F	OD: 14.5 OS: 16.7	OD:4.1 OS:3.9
AD 4	80	M	OD: 5.0	OD:6.9
AD 5	80	M	OS: 5.0	OS:11.5
AD 6	72	F	OD: 15.9 OS: 7.7	OD:12.1 OS:14.4
AD 7	90	F	OD: 5.6 OS: 13.0	OD:4.8 OS:5.0
AD 8	76	F	OD: 11.8	OD:12.1
AD 9	82	M	OD: 10.7 OS: 12.7	OD:17.0 OS:15.9
AD 10	81	M	OS: 9.6	OS: 9.6
AD 11	65	F	OD: 18.1 OS: 18.7	OD:9.4 OS:9.6
AD 12	66	M	OD: 10.0 OS: 17.0	OD: 4.7 OS:9.4
AD 13	76	M	OD: 14.1 OS: 17.7	OD:9.1 OS:7.5
AD 14	86	M	OD: 10.0	OD:8.8
Control 1	73	M	OD: 5.0 OS: 0.8	OD:3.5 OS:3.2
Control 2	62	M	OD: 10.4 OS: 2.6	OD:4.7 OS:4.7
Control 3	71	M	OD: 7.0 OS: 1.2	OD:7.4 OS:7.0
Control 4	71	F	OS: 4.6	OS: 3.6
Control 5	81	M	OD: 1.2 OS: 8.4	OD:4.4 OS:4.4
Control 6	89	F	OD: 3.5 OS: 3.0	OD:2.9 OS:2.3
Control 7	68	F	OD: 17.7 OS: 4.6	OD:7.4 OS:6.3
Control 8	66	F	OD: 3.2	OD:1.6
Control 9	65	F	OD: 6.6	OD:1.4

The eye from which the tear was collected is indicated. OD: Oculus dexter (right eye), OS: Oculus sinister (left eye).

**Fig 2:**
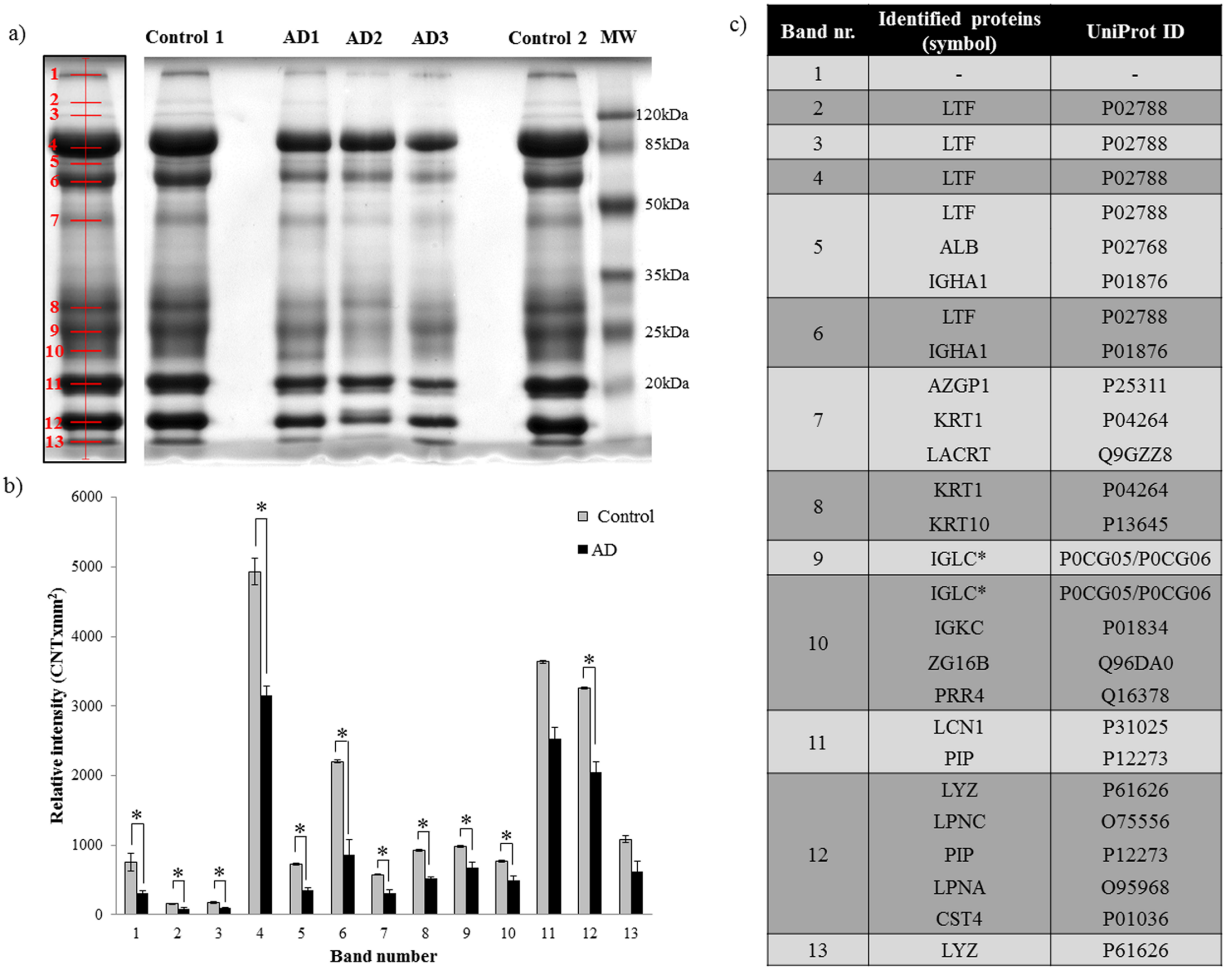
Changes in the tear proteome in AD. a) Gel image of tear proteins from three AD and two control subjects. The left panel shows the representative band distribution detected with QuantityOne. b) Densitometric analysis of gel bands. The bars show mean values with the standard error of mean, grey bars indicate control group while the black bars indicate the AD group. * indicates p <0.05. c) List of identified proteins with the corresponding UniProt reference numbers. * indicates the presence of Ig λ, though the Ig λ-2 chain C region and Ig λ-3 chain C region of the identified peptides were undistinguishable. LTF: lactotransferrin, ALB: serum albumin, IGHA1: Ig α-1 chain C region, AZGP1: Zn-α2 glycoprotein, KRT1: keratin, type II cytoskeletal 1, LACRT: extracellular glycoprotein lacritin, KRT10: Keratin, type I cytoskeletal 10, IGLC: Ig λ chain C region, IGKC: Ig κ chain C region, ZG16B: zymogen granule protein 16 homolog B, PRR4: proline-rich protein 4, LCN1: lipocalin-1, PIP: prolactin-inducible protein, LYZ: lysozyme-C, LPNC: lipophilin C, LPNA: lipophilin A, CST4: cystatin S.

Due to the limited amount of tear fluid; initially, tear profile changes were studied from a few randomly selected samples, thereafter, validation of the observed differences was carried out on a larger sample size. In order to monitor global tear protein changes characteristic to AD, equal amounts of tear proteins from three randomly selected AD patients and two controls were analyzed by electrophoresis. After visualization of protein bands and carrying out densitometric evaluation, 13 bands were observed in each case (Fig 2a), out of which 11 bands showed a significant decrease in band intensity in AD samples (Fig 2b). The bands were excised and digested using trypsin, followed by LC-MS/MS based protein identification (Fig 2c, S1 Table). Only those proteins; of which at least two peptides with ≥95% confidence could be detected, were accepted as present (S1 Table). Examining the differentially expressed proteins, we have identified them as being involved in the host defense, and are components of the chemical barrier of the eye. These data suggest that AD can alter the composition of the chemical barrier, this is in accordance with previous experiments showing alterations of the chemical barrier by different stimuli and pathological conditions [41–43].

### SRM-based quantitative proteomic method development for tear proteins

In order to validate changes of the chemical barrier in the tears of AD patients on a larger sample size, an SRM-based targeted proteomics approach was developed. The SRM scan mode provides a highly selective and sensitive method to monitor the level of multiple molecules in a single analysis; however, this method requires the proteins under examination to be defined priorly. For SRM assay design, lipocalin-1, lactotransferrin, lysozyme-C, extracellular glycoprotein lacritin, Ig λ, Zn α2 glycoprotein, prolactin inducible protein and lipophilin A proteins identified in bands showing significantly different intensity in AD samples as compared to controls were chosen. It is well known that the major proteins of sweat and tears play a role in host defense against potential pathogens [22,44], thus, based on previous experiments, galectin-3 binding protein and dermcidin; considered as proteins with an important role in host defense and immunomodulation [45,46], were also included into the panel of analyzed tear proteins.

SRM-based quantitative proteomic experiments were designed and optimized for the ten selected proteins (S2 Table). In order to enhance the specificity of the method, stable isotope-labeled (SIL) synthetic counterparts of the studied peptides were purchased and used in the analyses as an internal standard (S1 Fig). The sensitivity of the method was assessed by analyzing increasing amounts of SIL peptides added to the tear in a range of 10000 fold– 5 fold dilution. Most of the peptides were detectable in 10000 fold dilution (approximately 7.5 fmol), and the SRM signal intensity was proportional to the amount of peptides introduced in a broad range of dilutions (S3 Table). Based on our results, it appears that the developed SRM method is sensitive enough, and has a broad dynamic range enabling it to be a useful tool to monitor alterations of tear protein amounts.

### Changes in the tear chemical barrier`s composition in AD

The composition of the chemical barrier constantly changes upon different stimuli, as a response to mechanical and microbial challenges, in order to protect the human organism efficiently from potentially invading pathogens [47]. Moreover, systemic conditions can also affect the production and secretion of antimicrobial peptides, leading to changes in the composition of this antimicrobial peptide cocktail [47]. Tear fluid contains a variety of antimicrobial and immunomodulatory proteins as part of the first line defense of the innate immune system [22].

**Fig 3:**
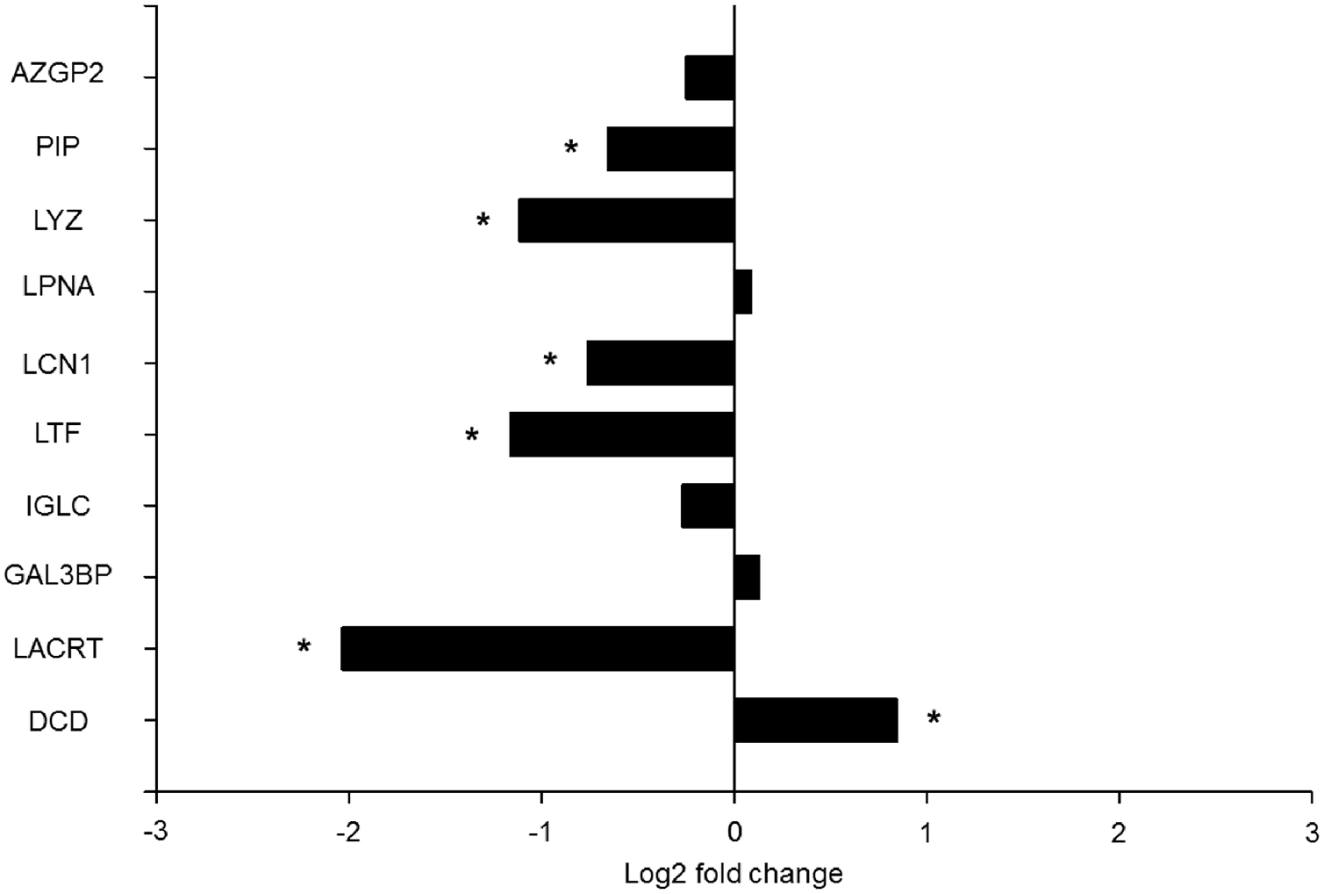
Quantitative analysis of tear proteins by SRM. The log2 fold change of the studied proteins in tears of patients with AD compared to controls. * indicate p≤0.05. AZGP1: Zn-α2 glycoprotein, PIP: prolactin-inducible protein, LYZ: lysozyme-C, LTF: lactotransferrin, LPNA: lipophilin A, LCN1: lipocalin-1, LACRT: extracellular glycoprotein lacritin, IGLC: Ig λ chain C region, GAL3BP: galectin 3-binding protein, DCD: dermcidin.

In order to study changes in the tear`s chemical barrier in AD, level of the ten selected proteins was analyzed using the developed SRM method in 37 tear samples from 14 patients with AD, and 9 controls in triplicates (S4 table). In line with results obtained from electrophoresis, the levels of lipocalin-1, lactotransferrin, extracellular glycoprotein lacritin, lysozyme-C, and prolactin inducible protein were significantly decreased, while the level of dermcidin was significantly elevated in AD tears, as compared to those of the controls (Fig 3,Table 2). The down-regulated proteins are expressed by the lacrimal glands, indicating lacrimal gland dysfunction in AD.

**Table 2:** Result of the mixed effect variance analysis.

Protein	Log2FC	SE	Tvalue	Adjusted p value
Zn-α2 glycoprotein	-0.25	0.17	-1.43	0.26
Prolactin-inducible protein	-0.66	0.14	-4.75	<0.0001
Lysozyme-C	-1.11	0.15	-7.29	<0.0001
Lipophilin A	0.09	0.23	0.39	0.76
Lipocalin-1	-0.76	0.13	-5.79	<0.0001
Lactotransferrin	-1.16	0.17	-6.66	<0.0001
Ig λ chain C region	-0.27	0.23	-1.19	0.35
Galectin 3-binding protein	0.13	0.19	0.68	0.59
Extracellular glycoprotein lacritin	-2.04	0.18	-11.53	<0.0001
Dermcidin	0.85	0.23	3.64	0.0006

The log2 fold change (log2FC) standard error (SE), T values (Tvalue) and the FDR corrected p-values are shown.

### Identification of potential AD-specific tear biomarkers

Proteins differentially expressed in the tears of AD patients were subjected to further analyses, in order to examine their potential use as future predictive biomarkers for AD. A receiver operator characteristic (ROC) analysis was carried out for each significantly expressed protein, and multivariate ROC curves were constructed to test the different combinations of potential biomarkers. The sensitivity, specificity, positive and negative predictive values and the area under the ROC curve (AUC) were calculated in each setting (Table 3). An AUC value close to 1.0 indicates a well-performing biomarker; whereas values close to 0.5 suggest a biomarker performing no better than random.

**Table 3:** ROC analysis of possible tear biomarkers for AD.

Proteins	Sensitivity (%)	Specificity (%)	PV+ (%)	PV- (%)	AUC	Accuracy	95% CI
LCN1	53.1	79.7	22.7	43.3	0.68	0.72	0.56–0.80
LTF	78.1	51.6	17.5	55.4	0.67	0.73	0.55–0.78
LYZ	90.6	50	8.6	52.5	0.68	0.67	0.57–0.79
PIP	71.9	56.2	20	54.9	0.6	0.67	0.48–0.72
LACRT	90.6	57.8	7.5	48.2	0.71	0.65	0.59–0.82
DCD	62.5	76.6	19.7	42.9	0.7	0.70	0.59–0.82
LCN1+DCD	53.1	89.1	20.8	29.2	0.74	0.76	0.63–0.85
LYZ+LACRT	90.6	59.4	7.3	47.3	0.72	0.64	0.61–0.83
LCN1+DCD+ LACRT	90.6	65.6	6.7	43.1	0.8	0.69	0.71–0.89
LCN1+DCD+LTF	53.1	89.1	20.8	29.2	0.74	0.76	0.62–0.84
LCN1+DCD+LYZ	59.4	82.8	19.7	36.7	0.75	0.76	0.64–0.86
LCN1+LTF+LYZ	87.5	50	11.1	53.3	0.72	0.71	0.61–0.83
LYZ+LACRT+ DCD	90.6	65.6	6.7	43.1	0.79	0.71	0.69–0.89
LYZ+LACRT+ LCN1	87.5	59.4	9.5	48.1	0.72	0.67	0.62–0.83
PIP+LACRT+DCD	93.8	60.9	4.9	45.5	0.79	0.68	0.69–0.97
DCD+LCN1+LTF+PIP	62.5	76.6	19.7	42.9	0.74	0.72	0.64–0.84
LCN1+DCD+LYZ+LACRT	81.2	76.6	10.9	36.6	0.8	0.72	0.71–0.89

In all cases, the AUC value was above 0.6, and when different combinations of two or more proteins were analyzed, AUC value was above 0.7, indicating an additive effect of the different proteins in improving the performance of the test. The highest sensitivity was obtained by combination of lysozyme-C and extracellular glycoprotein lacritin; yielding 91% sensitivity. The most balanced performance was achieved when lipocalin-1, dermcidin, lysozyme-C and extracellular glycoprotein lacritin were combined. In this case, the AUC was 0.80, the sensitivity was 81% and the specificity was 77%. Considering these values, it seems that a test combining these four potential biomarkers may therefore be used as a first screen in the diagnosis of AD.

## Discussion

Tear fluid is an excellent candidate for biomarker studies, given the facts that samples may be collected non-invasively, in addition to providing information not only on local ocular conditions; such as dry-eye disease and diabetic retinopathy, but also on systemic pathophysiological processes [22,25,29]. The presence of amyloid plaques was demonstrated in the retina and lens of patients with Alzheimer’s disease, and animal model studies indicated correlation in amyloid depositions between the retina and the brain [5,6]. Changes in the retinal vasculature and retinal morphology were detected in the eyes of patients with AD, resulting in the reduced visual performance observed in AD patients [13]. We have hypothesized that changes in retinal morphology and blood flow related to AD may alter the microenvironment of the eye, and this alteration may therefore be reflected at the level of tear proteins.

In our study, a significant increase in the flow rate and protein concentration, along with a significant difference in the amount of the studied tear proteins was observed in samples obtained from AD patients. This is in good agreement with previously published data suggesting extensive ocular alterations related to AD [5–7]. The protein level changes observed with electrophoretic analysis of five samples were validated by targeted proteomics analyses on 37 samples. The only increase in tear proteins characteristic to AD has been observed in case of dermcidin, which is produced by epithelial cells, [47] and is the main sweat antimicrobial peptide with a broad range antimicrobial activity [44]. The proteins with reduced amount were involved in first line defense of the eye, produced by the lacrimal gland.

The altered composition of the chemical barrier, along with the reduced level of studied defense proteins might imply an increased risk of ocular infections, yet there has been no reported increase of ocular infections in patients with AD according to the scientific literature. However, reduced corneal sensitivity and abnormal tear functions were reported in AD patients as compared to controls, in addition to other neurodegenerative diseases [48]. Dermcidin; with its broad antimicrobial spectrum, appears to be a plausible factor responsible for limiting bacterial over-growth and; therefore, possible infections.

Given the easy, non-invasive collection possibility of tears, the prospect of a bedside test development is worth exploring. Patients with an increased tear flow rate (above 5 μl/min according to our data), along with increased tear protein concentration, and who also show altered level of extracellular glycoprotein lacritin, lipocalin-1, lysozyme-C and dermcidin, may be subjected to further imaging, neuropsychological testing (CT, MRI, PET) and cerebrospinal fluid analyses (Aβ_42_, total tau and p-tau levels). Considering the small sample size analyzed in this pilot study, more studies carried out on a large population scale are required, in order to evaluate the applicability of the proposed biomarkers. After validation, tear analysis, as an easily executable test, can be used in population screening by general practitioners, and patients with a positive test may be further evaluated by clinical centers for the establishment of the diagnosis. If early diagnosis and treatment are promptly provided, the quality of life can be improved for AD patients and their caregivers, which will undoubtedly aid in decreasing the socio-economic burden of the disease.

## Supporting Information

S1 Fig Representative SRM spectra for each analyzed peptide. The y axis shows the intensity while the x axis shows the retention time. The blue line refers for the synthetic, stabile isotope labeled peptide, while the red line for the endogenous counterparts.
(PPTX)

S1 Table List of the identified proteins in tears. The name and accession numbers of the identified proteins in each band are presented along with the protein identification data. The sequence coverage (%Cov), the identified peptide sequences, the confidence of sequence identification (Conf), post-translational modifications (Modifications), cleavages, theoretical molecular weights and m/z values, the recorded precursor mass and m/z values, the delta mass (dMass) values are indicated along with the ID of spectrum used for sequence identification and retention time.
(XLSX)

S2 Table SRM transition parameters of examined tear proteins. Bold amino acids represent carbamidomethylated cysteines while * indicates the stable isotope-labeled amino acids. DP: de-clustering potential, CE: collision energy.
(DOCX)

S3 Table Linear dynamic range of the SIL peptides in tear matrix. The values represent the dilution range where the amount of peptide introduced into the mass spectrometer is proportional with the signal intensity.
(DOCX)

S4 Table AUC data determined by SRM analyzes from tears of AD patients and controls. The values of three independent replicates are indicated.
(XLS)
