# Supplementary material for: Changes in the Chemical Barrier Composition of Tears in Alzheimer’s Disease Reveal Potential Tear Diagnostic Biomarkers
Source: PLoS One. 2016 Jun 21;11(6):e0158000. doi: 10.1371/journal.pone.0158000 (PMC4915678; doi:10.1371/journal.pone.0158000)
Supplement: S1 Fig — The y axis shows the intensity while the x axis shows the retention time. The blue line refers for the synthetic, stabile isotope labeled peptide, while the red line for the endogenous counterparts. (PPTX) [file pone.0158000.s001.pptx]

## Slide 1
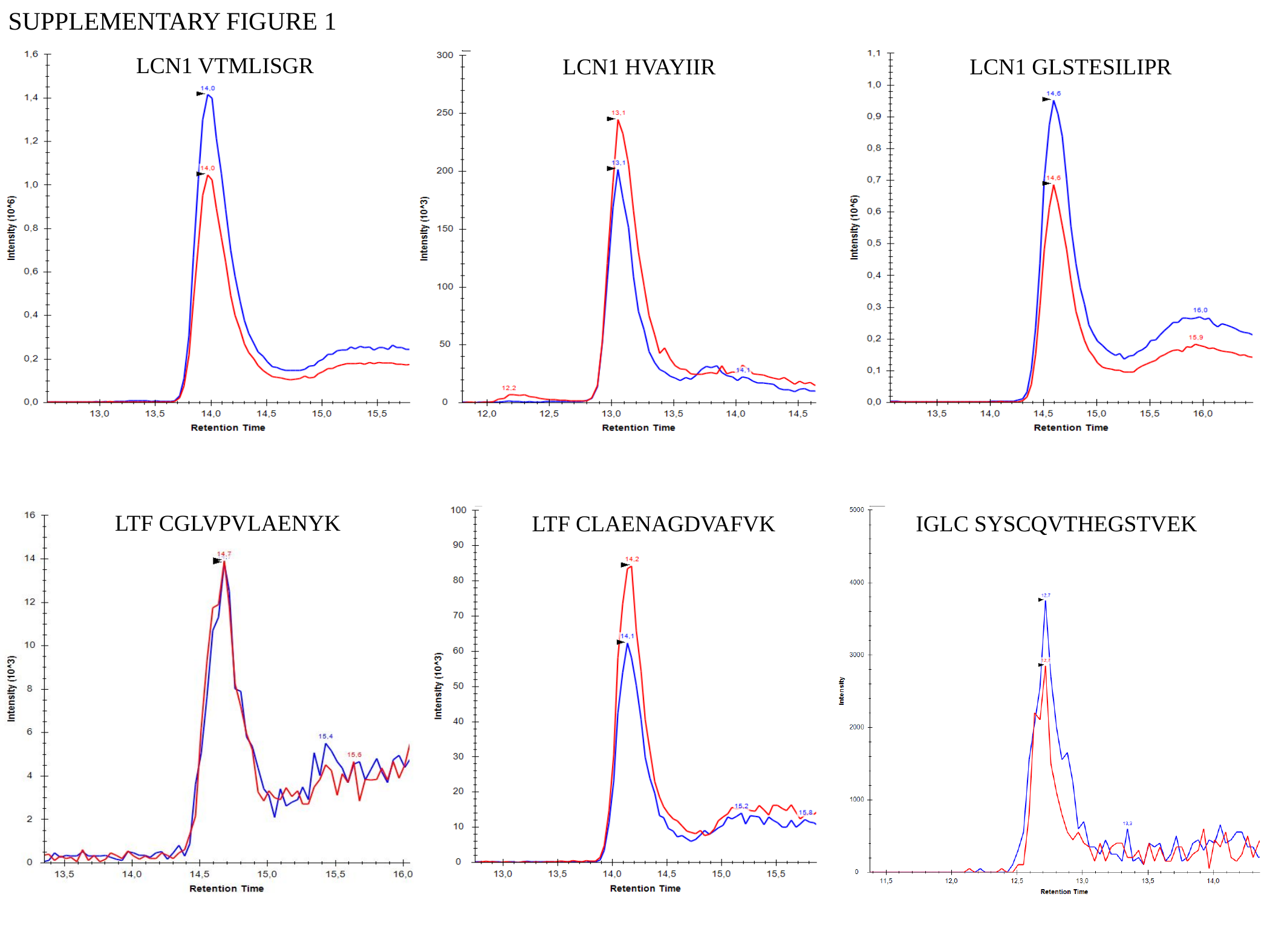

SUPPLEMENTARY FIGURE 1
LCN1 VTMLISGR
LCN1 HVAYIIR
LCN1 GLSTESILIPR
LTF CGLVPVLAENYK
LTF CLAENAGDVAFVK
IGLC SYSCQVTHEGSTVEK

## Slide 2
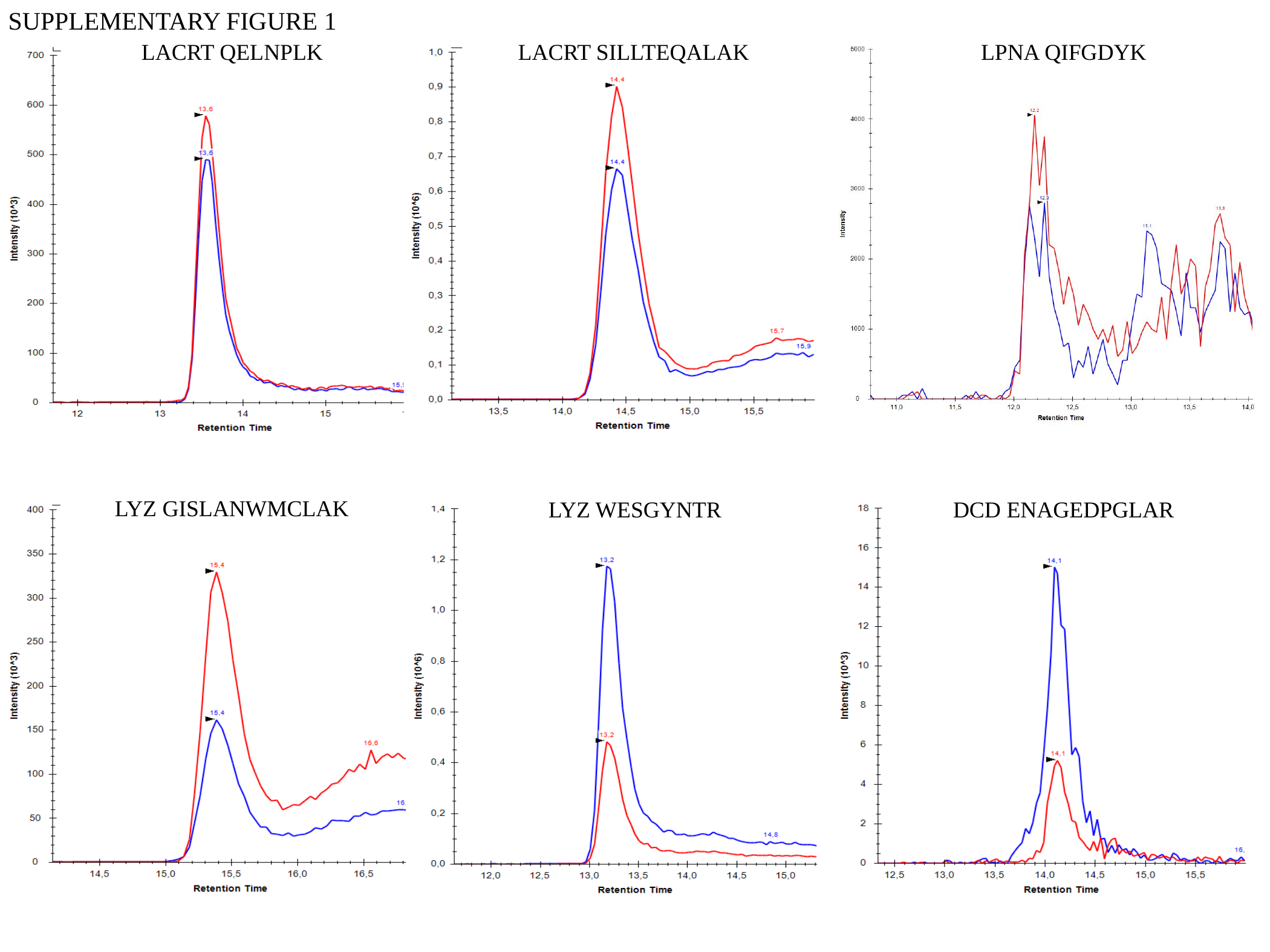

SUPPLEMENTARY FIGURE 1
LACRT QELNPLK
LACRT SILLTEQALAK
LPNA QIFGDYK
LYZ GISLANWMCLAK
LYZ WESGYNTR
DCD ENAGEDPGLAR

## Slide 3
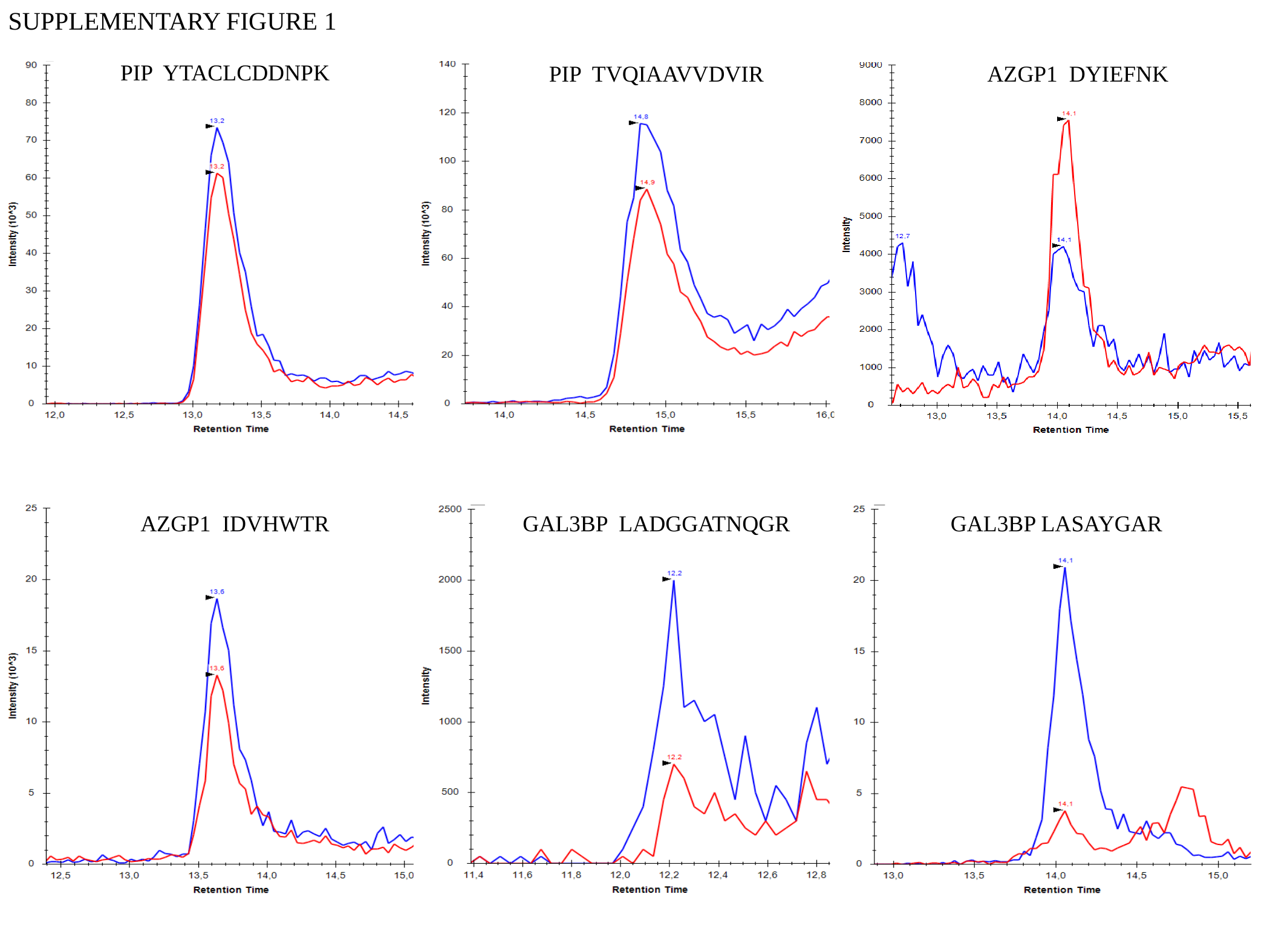

SUPPLEMENTARY FIGURE 1
PIP YTACLCDDNPK
PIP TVQIAAVVDVIR
AZGP1 DYIEFNK
AZGP1 IDVHWTR
GAL3BP LADGGATNQGR
GAL3BP LASAYGAR
