# Supplementary material for: Changes in the Chemical Barrier Composition of Tears in Alzheimer’s Disease Reveal Potential Tear Diagnostic Biomarkers
Source: PLoS One. 2016 Jun 21;11(6):e0158000. doi: 10.1371/journal.pone.0158000 (PMC4915678; doi:10.1371/journal.pone.0158000)
Supplement: S2 Table — Bold amino acids represent carbamidomethylated cysteines while * indicates the stable isotope-labeled amino acids. DP: de-clustering potential, CE: collision energy. (DOCX) [file pone.0158000.s003.docx]

Supplementary table 2: SRM transition parameters of examined tear proteins. Bold amino acids represent carbamidomethylated cysteines while * indicates the stable isotope-labeled amino acids. DP: de-clustering potential, CE: collision energy.

| **Protein** | **Peptide** | **Q1 (m/z)** | **Q3 (m/z)** | **Ion** | **Dwell time (msec)** | **DP (eV)** | **CE (eV)** |
| --- | --- | --- | --- | --- | --- | --- | --- |
| Lipocalin-1  (LCN1) | VTMLISGR | 438.752 | 676.381 | y6 | 20 | 63.1 | 20.7 |
|  |  |  | 432.257 | y4 | 20 | 63.1 | 20.7 |
|  | VTMLISGR* | 443.756 | 686.389 | y6 | 20 | 63.1 | 20.7 |
|  |  |  | 442.265 | y4 | 20 | 63.1 | 20.7 |
|  | HVAYIIR | 436.261 | 564.350 | y4 | 20 | 62.9 | 20.6 |
|  |  |  | 175.119 | y1 | 20 | 62.9 | 20.6 |
|  | HVAYIIR* | 441.265 | 574.359 | y4 | 20 | 62.9 | 20.6 |
|  |  |  | 185.127 | y1 | 20 | 62.9 | 20.6 |
|  | GLSTESILIPR | 593.346 | 498.340 | y4 | 20 | 74.4 | 29.6 |
|  |  |  | 272.172 | y2 | 20 | 74.4 | 29.6 |
|  | GLSTESILIPR* | 598.350 | 508.348 | y4 | 20 | 74.4 | 29.6 |
|  |  |  | 282.180 | y2 | 20 | 74.4 | 29.6 |
| Lactotransferrin  (LTF) | **C**GLVPVLAENYK | 681.858 | 737.383 | y6 | 20 | 80.8 | 34.6 |
|  |  |  | 553.262 | y4 | 20 | 80.8 | 34.6 |
|  | **C**GLVPVLAENYK* | 685.865 | 745.397 | y6 | 20 | 80.8 | 34.6 |
|  |  |  | 632.313 | y5 | 20 | 80.8 | 34.6 |
|  | **C**LAENAGDVAFVK | 697.343 | 920.484 | y9 | 20 | 82.0 | 35.5 |
|  |  |  | 735.404 | y7 | 20 | 82.0 | 35.5 |
|  | **C**LAENAGDVAFVK* | 701.350 | 928.498 | y9 | 20 | 82.0 | 35.5 |
|  |  |  | 743.418 | y7 | 20 | 82.0 | 35.5 |
| Extracellular glycoprotein lacritin  (LACRT) | QELNPLK | 421.243 | 584.377 | y5 | 20 | 61.8 | 19.7 |
|  |  |  | 357.250 | y3 | 20 | 61.8 | 19.7 |
|  | QELNPLK* | 425.250 | 592.391 | y5 | 20 | 61.8 | 19.7 |
|  |  |  | 365.264 | y3 | 20 | 61.8 | 19.7 |
|  | SILLTEQALAK | 593.856 | 873.504 | y8 | 20 | 74.4 | 29.6 |
|  |  |  | 760.420 | y7 | 20 | 74.4 | 29.6 |
|  | SILLTEQALAK* | 597.863 | 881.518 | y8 | 20 | 74.4 | 29.6 |
|  |  |  | 768.434 | y7 | 20 | 74.4 | 29.6 |
| Lysozyme-C  (LYZ) | GISLANWM**C**LAK | 682.347 | 993.464 | y8 | 20 | 80.9 | 34.6 |
|  |  |  | 922.427 | y7 | 20 | 80.9 | 34.6 |
|  | GISLANWM**C**LAK* | 686.354 | 1001.479 | y8 | 20 | 80.9 | 34.6 |
|  |  |  | 930.442 | y7 | 20 | 80.9 | 34.6 |
|  | WESGYNTR | 506.728 | 826.369 | y7 | 20 | 68.1 | 24.6 |
|  |  |  | 697.326 | y6 | 20 | 68.1 | 24.6 |
|  | WESGYNTR* | 511.732 | 836.377 | y7 | 20 | 68.1 | 24.6 |
|  |  |  | 707.335 | y6 | 20 | 68.1 | 24.6 |
| Lipophilin A  (LPNA) | QIFGDYK | 435.721 | 629.293 | y5 | 20 | 62.9 | 20.6 |
|  |  |  | 310.176 | y2 | 20 | 62.9 | 20.6 |
|  | QIFGDYK* | 439.729 | 637.307 | y5 | 20 | 62.9 | 20.6 |
|  |  |  | 318.190 | y2 | 20 | 62.9 | 20.6 |
| Ig λ-chain C region  (IGLC) | SYS**C**QVTHEGSTVEK | 856.383 | 987.474 | y9 | 20 | 93.5 | 44.5 |
|  |  |  | 276.155 | y2 | 20 | 93.5 | 44.5 |
|  | SYS**C**QVTHEGSTVEK* | 860.390 | 995.488 | y9 | 20 | 93.5 | 44.5 |
|  |  |  | 284.170 | y2 | 20 | 93.5 | 44.5 |
| Prolactin inducible protein  (PIP) | YTA**C**L**C**DDNPK | 678.782 | 1092.445 | y9 | 20 | 80.6 | 34.4 |
|  |  |  | 748.293 | y6 | 20 | 80.6 | 34.4 |
|  | YTA**C**L**C**DDNPK* | 682.789 | 1100.459 | y9 | 20 | 80.6 | 34.4 |
|  |  |  | 756.307 | y6 | 20 | 80.6 | 34.4 |
|  | TVQIAAVVDVIR | 642.388 | 842.509 | y8 | 20 | 77.9 | 32.4 |
|  |  |  | 771.472 | y7 | 20 | 77.9 | 32.4 |
|  | TVQIAAVVDVIR* | 647.392 | 852.518 | y8 | 20 | 77.9 | 32.4 |
|  |  |  | 781.481 | y7 | 20 | 77.9 | 32.4 |
| Zn α2 glycoprotein  (AZGP1) | DYIEFNK | 464.724 | 650.351 | y5 | 20 | 65.0 | 22.2 |
|  |  |  | 537.267 | y4 | 20 | 65.0 | 22.2 |
|  | DYIEFNK* | 468.731 | 658.365 | y5 | 20 | 65.0 | 22.2 |
|  |  |  | 545.281 | y4 | 20 | 65.0 | 22.2 |
|  | IDVHWTR | 463.746 | 599.305 | y4 | 20 | 64.9 | 22.2 |
|  |  |  | 462.246 | y3 | 20 | 64.9 | 22.2 |
|  | IDVHWTR* | 468.750 | 609.313 | y4 | 20 | 64.9 | 22.2 |
|  |  |  | 472.254 | y3 | 20 | 64.9 | 22.2 |
| Galectin 3 binding protein  (GAL3BP) | LADGGATNQGR | 530.263 | 575.290 | y5 | 20 | 69.8 | 26.0 |
|  |  |  | 175.119 | y1 | 20 | 69.8 | 26.0 |
|  | LADGGATNQGR* | 535.267 | 585.298 | y5 | 20 | 69.8 | 26.0 |
|  |  |  | 185.127 | y1 | 20 | 69.8 | 26.0 |
|  | LASAYGAR | 404.719 | 624.310 | y6 | 20 | 60.6 | 18.8 |
|  |  |  | 175.119 | y1 | 20 | 60.6 | 18.8 |
|  | LASAYGAR | 409.723 | 634.318 | y6 | 20 | 60.6 | 18.8 |
|  |  |  | 185.127 | y1 | 20 | 60.6 | 18.8 |
| Dermcidin  (DCD) | ENAGEDPGLAR | 564.768 | 628.341 | y6 | 20 | 72.3 | 27.9 |
|  |  |  | 513.314 | y5 | 20 | 72.3 | 27.9 |
|  | ENAGEDPGLAR* | 569.772 | 638.350 | y6 | 20 | 72.3 | 27.9 |
|  |  |  | 523.323 | y5 | 20 | 72.3 | 27.9 |
