# Supplementary material for: Changes in the Chemical Barrier Composition of Tears in Alzheimer’s Disease Reveal Potential Tear Diagnostic Biomarkers
Source: PLoS One. 2016 Jun 21;11(6):e0158000. doi: 10.1371/journal.pone.0158000 (PMC4915678; doi:10.1371/journal.pone.0158000)
Supplement: S3 Table — The values represent the dilution range where the amount of peptide introduced into the mass spectrometer is proportional with the signal intensity. (DOCX) [file pone.0158000.s004.docx]

Supplementary table 3: Linear dynamic range of the SIL peptides in tear matrix. The values represent the dilution range where the amount of peptide introduced into the mass spectrometer is proportional with the signal intensity.

| **Protein** | **Peptide** | **Linear dynamic range (dilution fator)** |
| --- | --- | --- |
| Lipocalin-1  (LCN1) | VTMLISGR | 10.000x-250x |
|  | HVAYIIR | 10.000x-5x |
|  | GLSTESILIPR | 500x-25x |
| Lactotransferrin  (LTF) | CGLVPVLAENYK | 500x-50x |
|  | CLAENAGDVAFVK | 500x-50x |
| Extracellular glycoprotein lacritin  (LACRT) | QELNPLK | 10.000x-500x |
|  | SILLTEQALAK | 100x-10x |
| Lysozyme-C  (LYZ) | GISLANWMCLAK | 5000x-25x |
|  | WESGYNTR | 500x-100x |
| Lipophilin A  (LPNA) | QIFGDYK | 10.000x-250x |
| Ig λ-chain C region  (IGLC) | SYSCQVTHEGSTVEK | 10.000x-100x |
| Prolactin inducible protein  (PIP) | YTACLCDDNPK | 10.000x-250x |
|  | TVQIAAVVDVIR | 10.000x-10x |
| Zn α2 glycoprotein  (AZGP1) | DYIEFNK | 10.000x-250x |
|  | IDVHWTR | 10.000x-100x |
| Galectin 3 binding protein  (GAL3BP) | LADGGATNQGR | 10.000x-250x |
|  | LASAYGAR | 10.000x-500x |
| Dermcidin  (DCD) | ENAGEDPGLAR | 10.000x-250x |
